# Comparison of Androgen Receptor, VEGF, HIF-1, Ki67 and MMP9 Expression between Non-Metastatic and Metastatic Stages in Stromal and Tumor Cells of Oral Squamous Cell Carcinoma

**DOI:** 10.3390/life11040336

**Published:** 2021-04-10

**Authors:** Lovorka Batelja-Vuletic, Cedna Tomasovic-Loncaric, Marcello Ceppi, Marco Bruzzone, Aleksandra Fucic, Karolina Krstanac, Vanja Boras Vucicevic

**Affiliations:** 1 Medical School, Zagreb, University of Zagreb, 10000 Zagreb, Croatia; lovorka.batelja.vuletic@mef.hr; 2 Clinical Hospital Centre Dubrava, 10000 Zagreb, Croatia; ctomasov@kbd.hr (C.T.-L.); kkrstanac@kbd.hr (K.K.); 3 IRCCS-Ospedale Policlinico San Martino, 16124 Genova, Italy; marcello.ceppi@hsanmartino.it (M.C.); marco.bruzzone@hsanmartino.it (M.B.); 4 Institute for Medical Research and Occupational Health, 10000 Zagreb, Croatia; 5 School of Dentistry, University of Zagreb, 10000 Zagreb, Croatia; borasvanja@yahoo.com

**Keywords:** oral squamous cell carcinoma, androgen receptor, VEGF, HIF-1, Ki67, MMP9

## Abstract

Objectives: Oral squamous cell carcinoma (OSCC) is the most common oral malignancy with low survival as it is very often diagnosed at an advanced stage, which is why the accurate profiling of the tumor is essential. The aim of this study was to, for the first time, compare in OSCC the frequency of AR, VEGF, MMP9, HiF1beta and Ki67 between the non-metastatic and metastatic disease. Materials and Methods: In the study, 96 non-metastatic and 91 metastatic OSCC patients were analysed for AR, VEGF, MMP9, HiF1beta and Ki67 levels by immunohistochemistry. Results: All of the tested biomarkers significantly differed between non-metastatic and metastatic disease. A significant association was found between >/=20% AR positive epithelium cells in cytoplasm, Ki67 and VEGF in cancer stroma. Ki67, HiF1beta, VEGF and MMP9 were significantly associated with TNM stages. Conclusion: Our results show for the first time an interplay between AR, VEGF, MMP9, HiF1beta and Ki67 in OSCC which may contribute to better diagnostics and therapy selection.

## 1. Introduction

The complexity of cancer mechanisms requires a multi-biomarker approach for improving diagnostics, therapy decisions and monitoring in personalized oncology. Oral squamous cell carcinoma (OSCC) is the most common, aggressive malignant epithelial neoplasm affecting the oral cavity, accounting for 2–4% of all cancer cases worldwide. Although the survival rates of OSCC have improved over the last two decades, the prognosis is still not satisfactory in comparison with therapy development and success achieved for other cancer types [1,2]. There is still no consensus on postoperative adjuvant therapy and criteria for high-risk diseases [3,4]. Prognostic factors are multiple and their interplay is complex and still unclear. Thus, there exists a significant need for a selection of biomarker batteries that could be used to improve diagnostics and determine the most effective treatment method [5].

Androgen receptor impact on neoplastic progress has drawn specific interest, as the significance of testosterone and estrogen axis was recognized in the etiology of all cancer types [6]. In the determination of an OSCC patient’s prognosis, in addition to the most well-known critical factors such as disease stage at initial diagnosis, tumor thickness, size, grade of cell differentiation and depth of invasion, insight into the interaction of AR positive cells in stroma and neoplastic epithelium with biomarkers associated with cancer progression are of crucial significance [7,8,9,10,11]. 

Vascular endothelial growth factor (VEGF) is over-expressed in OSCC and it is in the focus of interest in the new target drugs that are under development [1], as increased cell proliferation and vascularity exhibit interaction in malignant transformation [12]. 

Metalloproteinases assume a key role in the decomposition of extracellular matrix (ECM) by destroying the connective tissue matrix, resulting in tumor metastasis. Clinical studies have reported a high expression of MMP-9 in patients with OSCC [13]. Although not described in OSCC, VEGF expression has been positively linked with MMP-9 in stomach cancer [14].

Hypoxia is one of the hall-marks of cancer caused by the imbalance between oxygen consumption by rapidly proliferating cancer cells and insufficient blood supply. One mechanism against hypoxia in neoplastic tissue is the activation of hypoxia-inducible factor 1. Although the relationship between HIF-1a expression and tumor progression has been described in head and neck cancer [15,16], there is no data on HiF1 beta and its interaction with other biomarkers of OSCC progression. In some cancer types, VEGF, AR, and HIF-1 cross talk have already been recognized [17,18,19], similar to AR/MMP9/VEGF cross-talk [20]. 

The stroma has a significant part in cancer progression and its biology is of great interest but is still not always part of patho-histological diagnostics [21,22]. Stromal cells, cancer activated fibroblasts CAF in particular (CAF) and other cell types, such as endothelial cells, various immune/inflammatory cells, bone marrow–derived cells, adipocytes, pericytes and smooth muscle cells are in a network with cancer cells and their signaling interplay is crucial for growth promotion, invasiveness and the consequent metastatic spreads [23,24]. This signaling complex between the stroma and cancer cell is therefore interesting not only for its prognostic significance but also as a potential targeted therapy [25,26]. 

Currently, there is no data on the interaction of AR, VEGF, MMP9, HIF-1beta and Ki67 in OSCC stroma and neoplastic epithelium. Thus, the aim of this study was to compare the frequency of AR, HIF-1 beta, VEGF, Ki67 and MMP9 levels between non-metastatic and metastatic disease in the stroma and epithelium of OSCC. This study is based on previous results showing that the cut off value of 20% of AR positive cells in the cytoplasm of a neoplastic OSCC epithelium is a prognostic biomarker for a risk of metastasis [8].

## 2. Material and Subjects

In this study, 96 non-metastatic and 91 metastatic OSCC patients were analyzed. Stages of OSSC cancer ranged from T1N0 to T4N2. In patients considered to be in a non-metastatic stage, the mean age was 62.8y (males 71.9%), while in the patients with metastatic disease the mean age was 61.9y (males 86.8%). 

Patients were selected consecutively by date of hospitalization one after another without interruption. The inclusion criteria implied patients had not suffered suffer from any neoplastic disease before or at the time of OSCC diagnosis, that no distant metastases has been found at the time of OSCC diagnosis and that they had not been treated by radiation and/or antineoplastic drugs or hormonal therapy before. All of the performed procedures were in accordance with the ethical standards of the 1964 Declaration of Helsinki I. The study was approved by the Ethics Committee of the Clinical Hospital “Dubrava”, Zagreb, Croatia. The tissue specimens used in the current study were part of the hospital tissue archive. Patient consent was waived as the study used residual tissues from the archive of the Department of Pathology of Clinical Hospital Dubrava and biomarker data were analyzed with no associated identifiers.

Biopsy specimens were obtained after clinical diagnosis and surgical removal procedure of the primary OSCC and regional, cervical lymph nodes. Patients had not been treated before surgery (irradiation or chemotherapy). Immunohistochemistry was done on primary tumor samples. Metastatic patients only with local metastasis were included. Out of a total of 187 diagnosed cases of OSCC, 91 (48.7%) had metastases in the cervical lymph nodes at the time of diagnosis. There was no significant difference in age, sex and tumor localization distribution between the non-metastatic and metastatic OSCC patients. A description of the patients included in this study is presented in Table 1.

Resected tissue specimens were formalin-fixed, paraffin-embedded, and cut on microtome to form tissue sections (thickness 5 μm). Immunohistochemical analyses were performed after tissue section deparaffinization following microwave streptavidin immunoperoxidase protocol and using labelled streptavidin-biotin method on a DAKO autostainer with monoclonal antibodies for AR (clone AR441, M356201, DAKO, Glostrup, Denmark), Ki-67 (clone MIB-1, M724001, DAKO, Denmark), VEGF (clone VG1, M727329, DAKO, Denmark), MMP 9 (EP 1254, ab76003, Abcam, Cambridge, UK) and HF1beta (clone 2B10, ab 2771, Abcam, Cambridge, UK) Slides were counterstained with hematoxylin and eosin. Appropriate positive and negative controls were included in each immunohistochemistry run. Immunoreactivity reactions were determined in the cytoplasm and/or nucleus of neoplastic epithelium and stromal cells under a magnification of 400X for a total of 1000 tumor cells. Due to a high percentage of VEGF and MMP9 positive cells in stroma, for additional analysis of the stromal profile, macrophages and lymphocytes were scored separately. Discrimination between tumor epithelial cells and benign or tumor stroma and its cells was based on morphology and performed by experienced pathologist. Allred scoring was applied (https://medical-dictionary.thefreedictionary.com/Allred+scoring+system (accessed on 2 April 2021) using a cutoff value of <10% staining intensity. Analysis was performed by two experienced histopathologists (T.-L.C; B.-V.L.). Each biomarker was analysed by a single scorer on blinded slides. Quality control of analyses was achieved by supervision from an internal observer. Due to the high quality of immunostaining, a high reproducibility agreement was accomplished during internal quality control.

### Statistics

To investigate the relationship between AR, HIF-1 beta Ki67, MMP 9 and VEGF in epithelial and stromal cells with respect to the risk of the occurrence of metastases and TNM classification, a log-normal regression model was applied to the markers, adjusted by age and gender. By applying the same model, the predictability of the AR level in cytoplasm for metastatic and non-metastatic patients was also tested. This statistical model allowed us to estimate the Mean Ratio (MR) along with its 95% Confidence Interval (95% CI). MR is the ratio between the mean of the marker in a level of a predictor with respect to the level of the predictor taken as reference. MR is a dimensionless measure and expresses the percentage change in the frequency of the marker between the two levels of the predictor.

## 3. Results

**Figure 1:**
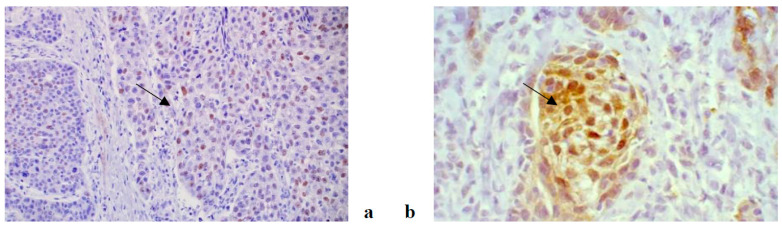
AR antibody, nuclear positivity in cancer cells in non metastatic OSCC (**a**) and cytoplasmic and nuclear positivity in cancer cells of metastatic OSCC (**b**). (magnification 200×).

**Figure 2:**
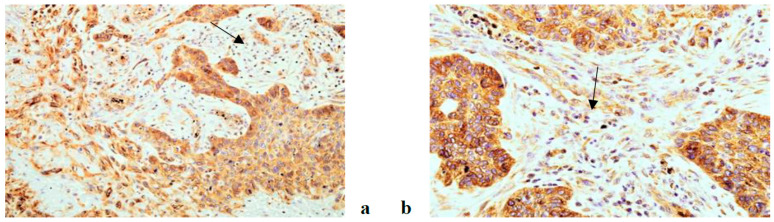
VEGF antibody, cytoplasmic positivity in cancer and stromal cells in non metastatic (**a**) and metastatic (**b**) OSCC. (magnification 200×).

**Figure 3:**
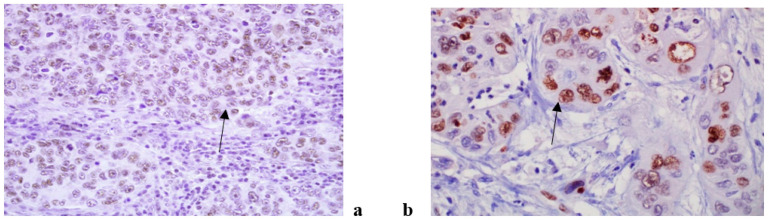
Hif 1 beta antibody, nuclear positivity in cancer cells in non metastatic (**a**) and metastatic (**b**) OSCC. (magnification 200×).

**Figure 4:**
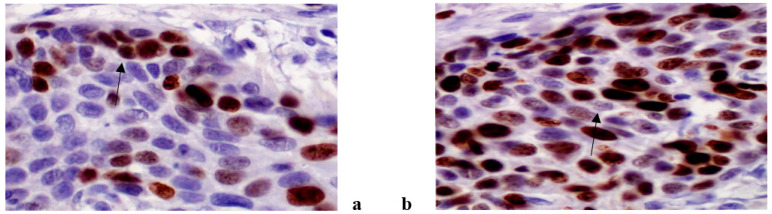
Ki 67 antibody, nuclear positivity in non metastatic OSCC (**a**) and metastatic OSCC (**b**). (magnification 400x).

**Figure 5:**
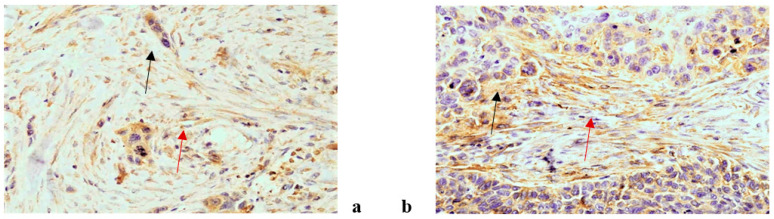
MMP9 antibody, cytoplasmic positivity in cancer (black arrow) and stromal cells (red arrow)of non metastatic OSCC (**a**) and in metastatic OSCC (**b**). (magnification 200×).

An immunohistochemical analysis of AR, Ki67, VEGF, HF1beta and MMP9 was performed in 187 patients suffering from OSCC (96 non-metastatic and 91 metastatic). The expression of all tested markers was present both in cancer and stromal cells with varying frequency. AR, Ki67 and HF1beta were more strongly expressed in cancer cells than in stroma, VEGF was almost equally expressed in both, while MMP9 was more strongly expressed in stroma than cancer cells. Representative immunohistochemistry characteristics of the studied markers are shown in Figure 1, Figure 2, Figure 3, Figure 4 and Figure 5. The results of the expression of the tested biomarkers in patients suffering from metastatic and non-metastatic OSSC are presented in Table 2. This table also reports the MR comparing the means of each biomarker in metastatic vs. non metastatic patients. As evident, all of the biomarkers significantly differed between non-metastatic and metastatic disease, some positively (MR > 1) others negatively (MR < 1). The expression of all of the tested biomarkers in cancer cells significantly differed between non-metastatic and metastatic disease, except for AR. A significant difference in AR, Ki67 and VEGF expression in cancer stroma between non-metastatic and metastatic disease was found. Due to a higher frequency of positive cells in stroma for VEGF and MMP9, further stratification was performed by scoring positive signals in macrophages and lymphocytes. Significantly more MMP9 positive stromal macrophages were detected in metastatic patients, while lymphocytes showed no significant difference contrary to VEGF for which significantly more positive macrophages were detected in non-metastatic patients.

Table 3 reports the biomarkers that exhibit a statistically significant relationship with respect to the frequency of cells with positive cytoplasmic AR and occurrence of metastasis. In metastatic patients, a significant increase of Ki67 positive cells in epithelium with a higher frequency of AR positive cells in the cytoplasm was detected. In metastatic diseases, HF1beta positive cells in the epithelium and VEGF positive macrophages have significantly lower levels independently of the percentage of AR positive neoplastic epithelial cells. Also in metastatic diseases, there is a significantly increased frequency of VEGF positive lymphocytes related to a cytoplasmic AR ≥ 20% and the frequency of MMP9 positive macrophages was significantly increased regardless of the percentage of AR positive neoplastic epithelial cells. 

A single case with HIF1beta immunohistochemical reaction limited to cytoplasm only was not detected. Very few cells with simultaneous nuclear and cytoplasmic positivity does not aloud any conclusion therefore we commented only on the nuclear positivity. The Ki67 frequency of positive cells in the stroma together with the HF1beta and VEGF frequency of positive cells in epithelium were significantly reduced in TNM 4, while the VEGF frequency of positive stromal lymphocytes and MMP9 positive stromal macrophages was significantly increased in TNM 3 and TNM 3 and 4, respectively. In TNM 3, there was a significant increase in MR for KI67 in epithelium in cases when the cytoplasmic AR was ≥20% (*p* = 0.013) (data not shown) (Table 4).

## 4. Discussion

Our study has for the first time shown a difference in AR, VEGF, MMP9, HiF 1beta and Ki67 positive cell levels in the stroma and neoplastic epithelium of OSCC between non-metastatic and metastatic disease. VEGF and Ki67 positive cells were shown to be significantly different between non-metastatic and metastatic disease both in stroma and epithelium. Frequency of HiF 1beta and MMP9 positive cells significantly differed between non-metastatic and metastatic disease in epithelium only and AR positive cells significantly differed between non-metastatic and metastatic disease in stroma only. VEGF positive stromal lymphocytes were shown to be significantly increased in case when cytoplasmic AR ≥ 20% of positive epithelium cells, the same as Ki67. An important finding was that a separate analysis of stromal macrophages and lymphocytes may be used for reaching a better understanding of cancer dynamics. As shown in our study, stromal macrophages are better biomarkers of cancer progression in case of MMP9. VEGF positive stromal lymphocytes and MMP9 positive macrophages had significant associations with TNM stages. The advantage of the applied MR as an effect estimate shows a positive or negative direction for investigated biomarkers with regard to metastasis. Our study for the first time shows the significant increase in MMP9 present only in metastatic patients, regardless of cytoplasmic AR levels. 

An integrated analysis of epithelium and stroma is of major importance as they both take part in tumorigenesis. Transformation of fibroblasts, various stem cells, immune cells, endothelial and even cancer cells into stromal CAF through the process known as endothelial/epithelial to mesenchymal transition (EMT) initiates the secretion of an entire spectrum of chemokines and cytokines promoting cancer invasion and metastasis [27,28]. Tumor activated macrophages (TAM) are the key inflammatory cells in the tumor stroma that secrete growth factors such as VEGF and MMP-9 with a role in the invasiveness and metastasis of tumor cells [29]. Cancer-associated fibroblast secrete proteolytic enzymes, which facilitate cell migration by degrading the extracellular matrix [24]. Some of the chemokines have chemotactic properties for endothelial progenitor cells and thus together with the secretion of VEGF, in which CAFs also participate, contribute to angiogenesis [30]. 

Androgen receptor plays a key role in the tumorigenesis of several malignancies as transcription factors [8,20,31,32]. Its overexpression is associated with poorer cell differentiation and contributes to the acquisition of EMT phenotype characteristics [31,32,33,34]. Additionally, the androgen induction of VEGF has been described in prostate cancer [35,36]. In a previous study [8], more than 20% of AR-positive cytoplasmic staining in the epithelium has been significantly associated with an increase in the AR nuclear positivity of the neoplastic epithelium and increased AR levels in stromal cells. Our results show that in metastatic disease AR ≥ 20% cytoplasmic positive neoplastic epithelial cells are significantly associated with the frequency of VEGF positive lymphocytes and Ki67. A similar finding was described in prostate and breast cancer [17,37].

Hypoxia inducible factor (HIF) is a transcription factor involved in carcinogenesis and tumor growth through the regulation of genes involved in angiogenesis, glycolytic metabolism and other biological mechanisms. The most studied factor is HIF-1, which in a tumor microenvironment foster the expression of VEGF [38,39]. Such a dynamic could be seen in our results, as a significant increase of HIF1ß expression in the epithelium and VEGF in stromal lymphocytes in TNM2 preceded a significant increase of VEGF in the epithelium in TNM3, thereby conditioning vascular supply development in a rapidly growing tumor mass. The increase of HIF 1 beta continues in TNM3. Similarly, to our results, the simultaneous upregulation of HIF-1α and MMP-9 was described in tumor tissues from patients with breast cancer. The underlying mechanism is suggested to be the fact that MMP-9 has more gelatinase activity under hypoxic than normoxic conditions [40].

The significant added value of this study is its analysis of lymphocytes and macrophages in stroma for VEGF and MMP9, which showed that these two cell types have a different dynamic, similar to that of a wound-healing process, as suggested by Karagiannis et al. 2012 [30]. Our results show that the expression of MMP9 in all stroma cell types does not differ between non-metastatic and metastatic disease. However, when the expression was analyzed in stromal macrophages and lymphocytes separately, a significant difference of expression of MMP-9 between non-metastatic and metastatic disease was found for macrophages. These finding suggests that the presence of a high frequency of MMP9 macrophages in stroma may be used as a prognostic biomarker as cancer tissues with high infiltration of tumor-associated macrophages are associated with poor patient prognosis and resistance to therapies [41,42].

The inclusion of TNM stages additionally clarified the interplay between the applied biomarkers. Thus, although MR was shown to be positive for VEGF lymphocytes and MMP9 macrophages between non-metastatic and metastatic disease, it was clear that the increase was most pronounced for TNM3. Similarly, although a significant increase in Ki67 in the epithelium associated with AR is present in metastatic disease, stromal Ki67 significantly decreased in TNM4. This could be associated with the observed replacement of stroma by acellular, collagen extracellular matrix in a more advanced stage of the disease [30]. 

In conclusion, the location, grade and stage of OSCC is crucial in diagnostics, selection of therapy and prognosis of survival. This study suggests that the profiling of tumor-based interaction between AR, ki67, VEGF, HIF1beta and MMP9 in epithelium and stroma may be a significant contribution in the personalized diagnostics of OSSC. These biomarkers complemented to TNM staging may become a significant tool in decision regarding therapy and frequency of medical checks after the completion of therapy. Another significant finding is that stromal lymphocytes and macrophages should be analyzed separately in order to achieve their applicability for further investigations or for clinical use.

## Figures and Tables

**Table 1:** Baseline characteristics of the patients with a tumor stage.

	Non Metastatic 96 Patients				
Age Years (mean)	Sex (%)	Tumor Localization (%)	Cervical Lymph Node Resection (%)	pT (%)	Stage Grouping pTNM (%)
62.8	Male 71.9 Female 28.1	Alveola ridges 31.1 Hard palate 6.9 Retromolar 13.8 Buccal mucosa and bucco alveolar sulci 24.1 Floor of mouth 24.1	No-node resection 36.5 node resection 63.5	T1 19.8 T2 42.7 T3 10.4 T4 27.1	I 11.5 II 50.8 III 8.2 IVa 29.5
	**Metastatic 91 patients**				
**Age Years** **(mean)**	**Sex** **(%)**	**Tumor Localization** **(%)**	**Cervical Lymph Node Resection (%)**	**pT** **(%)**	**Stage Grouping pTNM** **(%)**
61.9	Male 68 Female 32	Alveolar ridges 37.9 Retromolar 6.9 Buccal mucosa and Bucco alveolar sulci 20.7 Floor of mouth 34.5	No-node resection 0 node resection 100	T1 12.1 T2 34.1 T3 17.6 T4 6.2	III 20.9 IVa 76.9 VIb 29.5

**Table 2:** Description of the markers considered and their relationship with the occurrence of metastasis.

Biomarker	Non Metastatic N = 96, Mean ± SD	Metastatic N = 91, Mean ± SD	MR *	95% CI	*p*
**AR**					
cytoplasm	14.4 (20.1)	17.5 (19.7)	1.55	0.87–2.73	0.134
epithelium	3.3 (6.7)	5.7 (10.8)	1.44	0.97–2.12	0.070
stroma	1.8 (4.5)	4.5 (8.9)	1.48	1.01–2.18	**0.047**
**ki67**					
epithelium	45.9 (14.7)	50.1 (15.4)	1.10	1.01–1.21	**0.041**
stroma	14.3 (7.5)	12.2 (8.2)	0.78	0.64–0.95	**0.013**
**HF1beta**					
epithelium	26.6 (29.1)	14.3 (21.6)	0.37	0.21–0.65	**0.001**
stroma	11.0 (19.2)	6.4 (14.9)	0.93	0.57–1.51	0.760
**VEGF**					
epithelium	15.5 (17.4)	9.6 (15.1)	0.48	0.29–0.80	**0.005**
stroma	16.5 (22.0)	9.5 (12.8)	0.57	0.34–0.97	**0.036**
macrophages	15.7 (22.4)	5.5 (9.7)	0.41	0.24–0.69	**0.001**
lymphocytes	0.8 (4.4)	3.8 (10.4)	1.49	1.07–2.08	**0.019**
**MMP9**					
epithelium	18.9 (23.4)	15.2 (25.7)	0.54	0.30–0.98	**0.044**
stroma	39.1 (22.1)	43.6 (25.0)	1.22	0.88–1.69	0.240
macrophages	11.3 (15.5)	15.4 (14.2)	2.43	1.45–4.06	**0.001**
lymphocytes	27.5 (24.7)	29.3 (24.7)	1.17	0.58–2.33	0.662

* adjusted by age and gender; *p*-value < 0.05 (presented on bold) considered statistically significant.

**Table 3:** Markers that exhibit a statistically significant relationship with respect to the level of cytoplasmic AR and occurrence of metastasis.

	Mean Ratio [95% CI)		
	Cytoplasmic AR/Metastatic stage ª		
	>=20%/No	<20%/Yes	>=20%/Yes
Ki67 epithelium	0.89 (0.78–1.03)	1.01 (0.90–1.14)	**1.14** * (1.01–1.30)
HF1beta epithelium	1.01 (0.44–2.34)	**0.35** * (0.17–0.71)	**0.41** * (0.19–0.88)
VEGF macrophages	0.52 (0.23–1.16)	**0.36** * (0.19–0.71)	**0.32** * (0.16–0.63)
VEGF lymphocytes	1.35 (0.82–2.24)	1.50 (0.98–2.29)	**1.80** * (1.15–2.80)
MMP9 macrophages	1.62 (0.75–3.51)	**3.01** * (1.57–5.78)	**2.52** * (1.28–4.97)

ª reference level < 20%/No; * *p* < 0.05 (presented in bold); adjusted by age and gender.

**Table 4:** Markers that exhibit a statistically significant relationship with respect to TNM classification.

Biomarker	Mean Ratio [95% CI)		
	TNM ª		
	2	3	4
Ki67 stroma	0.99 (0.75–1.32)	1.09 (0.79–1.51)	**0.72** * (0.57–0.92)
HF1beta epithelium	0.49 (0.22–1.12)	**0.18** * (0.07–0.46)	**0.37** * (0.18–0.74)
VEGF epithelium	1.29 (0.63–2.66)	0.95 (0.42–2.15)	**0.45** * (0.24–0.83)
VEGF lymphocytes	0.87 (0.54–1.41)	**2.27** * (1.33–3.88)	1.16 (0.78–1.74)
MMP9 macrophages	1.27 (0.61–2.66)	**3.29** * (1.42–7.63)	**1.93** * (1.02–3.63)

ª reference level TNM 1; * *p* < 0.05 (presented in bold); adjusted by age and gender.

## Data Availability

The data presented in this study are available on request from the corresponding author. The data are not publicly available due to still ongoing analysis.
